# A whole-genome sequencing dataset of nanopore raw signals for bacterial genotyping and methylation analysis

**DOI:** 10.1038/s41597-025-06319-4

**Published:** 2025-12-02

**Authors:** Johanna Dabernig-Heinz, Valentina Galeone, Somayyeh Sedaghatjoo, Ivo Steinmetz, Christian Kohler, Martin Hölzer, Gabriel E. Wagner

**Affiliations:** 1 Diagnostic and Research Institute of Hygiene, Microbiology and Environmental Medicine, Medical University of Graz, Neue Stiftingtalstraße 6, 8010 Graz, Austria; 2 Genome Competence Center (MF1), Robert Koch Institute, Nordufer 20, 13353 Berlin, Germany; 3 Friedrich Loeffler Institute for Medical Microbiology, F.-Sauerbruch-Str., 17475 Greifswald, Germany

**Keywords:** Bioinformatics, Bacterial genetics, Bacterial infection, Genetics research, DNA methylation

## Abstract

This dataset comprises raw signal data from a multicenter study evaluating the accuracy of bacterial whole-genome genotyping using Oxford Nanopore long-read sequencing. The raw data comprises 79 isolates across six bacterial species, including 12 triplicates from three different laboratories (totalling ~1.4 TB of data). Sequencing was conducted on the latest R10.4.1 flow cells with V14 chemistry, producing on average 16 gigabases per flow cell. The generated raw ion current signals retain information beyond nucleotide sequences, supporting in-depth reanalysis for nucleotide modifications, resistance genes, and bacterial strain differentiation. The dataset enables re-basecalling with future models to keep up with the newest developments, e.g. to mitigate methylation-based calling errors, enhancing the reliability of SNP profiling and cgMLST analyses crucial for genomic surveillance. By sharing this raw signal data, accompanied by additional phenotypic resistance-data and an extensive quality control pipeline, we aim to advance reproducibility, support error correction studies and the continued development of bioinformatics tools, and encourage sharing raw data for broader genomic and epigenetic investigations as general best practice.

## Background & Summary

Genomic surveillance of pathogens is crucial for tracking outbreaks^1^, monitoring antimicrobial resistance markers^2^, and informing public health interventions^3,4^. In the context of bacterial pathogens, nanopore sequencing has become a widely used tool for whole-genome sequencing and assembly, offering unprecedented accessibility through its portability, speed, and lower initial cost barriers while allowing researchers to obtain complete genomes with high accuracy and contiguity^5–8^. This capability is precious for applications in genomic surveillance, such as plasmid identification^9,10^, resistance gene annotation^11^, SNP profiling, and core genome multilocus sequence typing (cgMLST), the latter being critical for tracking bacterial outbreaks and understanding pathogen evolution in public health and surveillance contexts^12,13^. While the basecalled data in the form of FASTQ files, containing DNA sequences and the associated quality scores, is regularly shared among researchers on platforms such as the National Center for Biotechnology Information’s Sequence Read Archive^14^ (NCBI SRA) and the European Nucleotide Archive^15^ (EMBL-EBI ENA), raw signal data in the form of FAST5 or POD5 files (also called “squiggle” data) is rarely exchanged despite the advantages discussed below. The raw signal data may only be stored temporarily after basecalling, given the challenges of enormous file sizes, impracticalities in data upload, and the need for tailored data-sharing platforms that emphasize the importance of researchers also sharing their squiggles. Here, we share a comprehensive dataset of squiggle data from 79 bacterial isolates sequenced across three laboratories using Oxford Nanopore Technology (ONT). Our dataset enables researchers to explore signal-level analysis, develop improved algorithms, and optimize bioinformatic pipelines for applications beyond bacterial genotyping.

The complexity of the raw signal data is rooted in the nanopore sequencing mechanism, which generates long sequencing reads by measuring disturbances in the ion current as biological molecules such as DNA and RNA pass through a nanopore^16,17^. The recorded raw time-series current, the squiggle, is the unprocessed electrical signal output from ONT sequencing devices. During or after sequencing, the raw DNA/RNA signal can be transformed into nucleotide sequences through basecalling algorithms such as those implemented in ONT’s Dorado basecaller. A vital characteristic of the squiggle data is that it is shaped by any molecular feature that affects the flow of electric current through the nanopore and the speed at which the molecule moves through the pore (the translocation speed)^16^. Thus, nanopore sequencing can detect biological features beyond the mere stage of primary nucleotide composition in DNA by reading native RNA^18^, protein sequences^19,20^, various DNA modifications^21^ and RNA^22^ signals, such as those caused by methylation, secondary RNA structures^23^, and even whether a DNA belongs to a dead or living bacterium^24^. However, with all these technological possibilities comes a particular challenge of correctly translating the squiggle signal into the biological features of interest^25,26^.

The squiggle data represents a treasure trove of information that we are just beginning to utilize. Sharing raw signal data alongside FASTQ files also enhances the reproducibility of results. Furthermore, with the frequent updates to nanopore’s basecalling models, only access to the raw signal data allows for reanalysis using the latest and more accurate algorithms to improve overall read accuracy or to detect nucleotide modifications.

One specific example highlighting the importance of sharing squiggle data is methylation-based basecalling errors, which can impact bacterial genome reconstruction and subsequent genotyping. While nanopore sequencing has shown impressive capabilities across various applications, such strain-specific challenges related to miscalling methylated bases currently limit its applicability for high-resolution bacterial genotyping, as we and others have reported in recent studies^26–29^. Even a few incorrectly basecalled nucleotides can heavily impact the accuracy of SNP-based genotyping methods, which can lead to the misclassification of outbreaks in clinical settings. However, introducing the new v5 Dorado basecalling models, combined with a tailored Medaka v2 model^30^ trained specifically for bacterial methylation^31^, significantly enhanced genotyping accuracy, pointing toward a viable solution. In our multicenter performance study^29^, nanopore sequencing had already demonstrated robust and consistent results across participants with non-problematic strains; thus, further advances in basecalling approaches suggest it may also meet routine surveillance demands soon for problematic strains. In a follow-up study, we demonstrated that newer basecalling models reduce strand-specific ambiguity, although some errors persist for specific motifs^32^. Most importantly, re-assessing nanopore sequencing data in such a way, as described here but especially for future updates, is only possible if the raw signal data is shared.

Advancing beyond our initial investigation^29^ and the field’s standard practice of providing only base-called reads, we present comprehensive nanopore raw signal data that significantly extends our previous analysis of ONT accuracy and reproducibility in bacterial pathogen genotyping. This comprehensive whole-genome sequencing dataset offers unprecedented access to signal-level information, enabling e.g. enhanced methylation analysis and pathogen characterization previously unattainable through conventional approaches. We additionally include newly derived phenotypic metadata on the antimicrobial resistance profiles of all strains of clinically relevant species, which facilitate integrative analysis of phenotype and genotype. The raw data files shared here were sequenced by three laboratories using the latest R10.4.1 flow cells, V14 chemistry at the default translocation speed of 400 bp/s. In total, the shared data set comprises six publicly relevant bacterial species totalling 79 biosamples and 102 individual SRA runs based on the current default translocation speed (400 bp/s). This allows extensive re-analysis, for example, to assess error rates and to detect methylation signals. Here, we demonstrate how this data set can be re-basecalled and utilized to reduce methylation-induced errors in the generated assemblies, thus improving cgMLST analyses. We further demonstrate the reproducibility of methylation detection analysis in our dataset by focusing on triplicate samples, showing that similar methylation levels can be consistently detected in the same isolates when sequenced at different laboratories. At the same time, we also enable its use in training machine learning models, advancing algorithm development, and even supporting education in nanopore sequencing and methylation analysis, particularly given the limited availability of such open and comprehensive raw signal squiggle datasets.

Despite their importance for signal-level analyses amongst others, FAIR principles are often neglected for nanopore raw data due to data volume and lack of suitable repositories. The demand for appropriate infrastructure is reflected in the recent development of Squidbase (https://docs.squidbase.org/), specifically designed for nanopore squiggle data, alongside continued efforts to develop optimized storage formats such as SLOW5^33^. With our comprehensive dataset including replicates, we make an important contribution toward making these valuable raw data comprehensively and sustainably available to the scientific community, contributing to the goal of a broader adoption of best practices for sharing nanopore raw data.

## Methods

### Strain selection and DNA isolation

We selected strains from six public-health-relevant bacterial species predominantly from Germany and Austria, collected between 2019–2022 from human hosts, to obtain a diverse collection of different sequence types^29^. These isolates comprise one *Enterococcus faecalis* (*El*), 19 *Enterococcus faecium* (*Ef*), 20 *Klebsiella pneumoniae* (*Kp*), 20 *Listeria monocytogenes* (*Lm*), 18 *Staphylococcus aureus* (*Sa*), and one *Staphylococcus simulans* (Ss) isolate, totaling in 79 different bacterial strains. A subset of 12 strains from four different species was selected based on previous results^29^ for sequencing in two additional laboratories to test the reproducibility of nanopore sequencing **(**Table 1). For these 12 strains, each laboratory had received identical pure cultures in stitch-agar propagated from a single colony in a blind-coded manner and had continued with cultivation and DNA preparation according to their protocols (Table 2). Since the study utilized only anonymized bacterial cultures with no connection to patient data and no additional specimens were collected beyond routine clinical care, the Ethics Committee of the University Medicine Greifswald confirmed that formal ethics approval was not required for this study.

**Table 1:** Overview of cultivation, DNA preparation protocols and number of sequenced isolates per laboratory.

	Cultivation			DNA preparation		Number of sequenced isolates					
	Medium	Duration	Temp.	Isolation	Single col.	*El*	*Ef*	*Kp*	*Lm*	*Sa*	*Ss*
LAB1	COL-S	24 h	37 °C	NucleoSpin + AXP Wash	yes (culture)	—	3	3	3	3	—
LAB2	COL-S	24 h	37 °C	NucleoSpin + AXP Wash	yes (culture)	1	19	20	20	18	1
LAB3	BHI	16 h @ 160 rpm	37 °C	MagAttract/ GenElute	yes (inoculation)	—	3	3	3	2*	—

The cultivation media were either solid Colombia sheep blood agar plates (COL-S) or liquid brain heart infusion (BHI) incubated at 37 °C overnight. The latter cultivation was performed in shakers for liquid media. Kits for DNA preparation/isolation were utilized according to the manufacturer’s instructions: NucleoSpin Microbial DNA (Macherey Nagel) and subsequent AXP Wash (Beckman Coulter) with a magnetic bead cleanup or MagAttract HMW DNA Kit Qiagen (in LAB3 for all species except *Lm*) and GenElute™ Bacterial Genomic DNA Kit (in LAB3 only for *Lm*). DNA isolation was performed from single-colony or liquid cultures. The species of the sequenced isolates are abbreviated as follows: *El* - *Enterococcus faecalis*, *Ef* - *Enterococcus faecium*, *Kp* - *Klebsiella pneumoniae*, *Sa* - *Staphylococcus aureus*, *Ss* - *Staphylococcus simulans*. 2*: Raw sequencing data for the third *Sa* isolate of LAB3 is unavailable due to unresolved technical issues during SRA deposition that persisted despite collaborative troubleshooting with NCBI support.

**Table 2:** Summary of nanopore sequencing runs for the raw signal files uploaded in this data descriptor.

	Nanopore sequencing (SQK-NBD114.24, R10.4.1, 400 bp/s, 5 kHz)								
	DNA amount loaded (ng)	Samples per run	MinKNOW version	Average pores before sequencing	Number of used flow cells	Data produced (GB)	Bases sequenced (Gb)	N50 (kb)	Fail rate (%)
LAB1	150	12	23.11.4	1140	1	174.98	15.05	12.6	18.4
LAB2	90	16–20	23.04.5	1447 ± 73	5	997.23	58.27	8.3 ± 1.2	33.6 ± 8.7
LAB3	82	12	23.07.12	1398	1	266.15	21.68	5.1	18.8

For LAB2, the values are either averages with standard deviations, or sums of all five runs (congruent with the number of used flow cells). The total of produced data per lab in gigabyte (GB) summarizes all files created during sequencing, including all POD5 files in passed and failed (and potentially skipped) folders, as well as basecalled FASTQ files and reports. The number of bases sequenced (in gigabases - Gb) correlates with the total data produced. Of all sequenced bases, a certain percentage is classified as failed based on, for example, low-quality base scores (noted in the fail rate).

The concentration of high-purity DNA from the respective preparation kits was measured with Qubit4 or Qubit Flex devices using the 1xds DNA BR kit or the 1xds DNA HS kit, depending on the expected DNA content.

### Phenotypic antimicrobial resistance testing

The pure cultures of the strain collection of three clinically relevant species (*Ef*, *Kp*, *Sa*) were also analyzed in a fully automated VITEK® 2 XL instrument (bioMérieux, France) in LAB1 for phenotypic identification of antimicrobial susceptibility. Depending on the species the following card types were used, each incorporating 21 to 26 different antibiotic tests suitable for the respective species (AST-P655 for *Enterococcus faecium*, AST-N433 for *Klebsiella pneumoniae*, AST-P654 for *Staphylococcus aureus*) using the VITEK 2 Systems Version 9.03.3 and interpretation following the EUCAST 2023 guidelines.

### Library preparation and nanopore sequencing

The library preparation was carried out in the same way in all laboratories using the ONT Native Barcoding Kit 24 V14 and following the protocol for gDNA ligation sequencing (SQK-NBD114.24). All sequencing was performed on R10.4.1 flow cells on MinION or GridION devices (Table 2). A set of 16–20 strains of the same species, or 12 strains from different species, to generate the sequencing triplicates, were sequenced on a single flow cell at the default translocation speed of 400 bp/s (5 kHz).

### Contextualization of 400 bp/s signal data in relation to the previous 260 bp/s performance study

Building upon our previous performance study of 79 diverse bacterial strains sequenced across five laboratories^29^, we now provide the underlying raw nanopore signal data (squiggles). While FASTQ files were previously released (but based on now outdated basecalling models), the raw signal data presented here unlock advanced applications and (re-)analysis beyond traditional basecalling, including novel algorithm development and methylation analysis. Supplementary Table 1 provides comprehensive sequencing statistics that, while based on the original dataset, were exclusively compiled for this release, analogous to Table 2. This release provides, for the first time, access to raw squiggle data generated exclusively at the standard 400 bp/s translocation speed. Given that the legacy 260 bp/s mode has been deprecated and the resulting data are incompatible with contemporary basecalling algorithms and analytical pipelines, these obsolete raw signal data is excluded from this data descriptor. Of note, squiggle data is now typically saved as POD5 files during sequencing rather than in FAST5 format. However, we have to provide the raw signal data as basecalled FAST5 files due to the current limitations of SRA, which does not support POD5 data files (personal communication). Thus, the raw signal files must be subsequently converted to the original POD5 format and merged into a single file per barcode using a command from the POD5 file format software provided by ONT, as explained in detail in the Usage Notes section below.

## Data Records

This data descriptor includes files representing direct whole-genome sequencing squiggle data of DNA isolations from pure bacterial cultures. The specific POD5/FAST5 file format contains the raw electrical signal measured in the nanopores with a recording speed of 400 bases per second sampled at 5 KHz. The main advantage of this raw data format is that the data is given the most significant possible reusability, as subsequent basecalling is possible with any available model suitable for the flow cell and translocation speed used. By default, this data is not often shared due to the large data storage requirements and the manual preparation required for a successful upload to common repositories. However, the availability of the raw squiggle data is the only way to keep the sequencing data up-to-date and usable for software updates.

For optimal data accessibility, Table 3 summarizes essential parameters for each squiggle data file provided with this data descriptor. For each isolate per laboratory, the table contains the library ID, i.e. the name by which individual files can be located online, the species, the size of each individual file, and the specific accession number.

**Table 3:** Newly published squiggle data from six different bacterial species^34^.

Library ID	Laboratory	Species	Size (GB)	SRA accession
EF21-PS_Lab2-raw	LAB2	*Enterococcus faecalis*	12	SRR32250977
EF22-PS_Lab1-raw	LAB1	*Enterococcus faecium*	16	SRR31990277
EF22-PS_Lab2-raw	LAB2	*Enterococcus faecium*	12	SRR32250976
EF22-PS_Lab3-raw	LAB3	*Enterococcus faecium*	1.8	SRR31990273
EF23-PS_Lab2-raw	LAB2	*Enterococcus faecium*	7.9	SRR32250965
EF24-PS_Lab2-raw	LAB2	*Enterococcus faecium*	13	SRR32250954
EF25-PS_Lab2-raw	LAB2	*Enterococcus faecium*	12	SRR32250943
EF26-PS_Lab1-raw	LAB1	*Enterococcus faecium*	12	SRR31990276
EF26-PS_Lab2-raw	LAB2	*Enterococcus faecium*	9.9	SRR32250932
EF26-PS_Lab3-raw	LAB3	*Enterococcus faecium*	4.1	SRR31990272
EF27-PS_Lab2-raw	LAB2	*Enterococcus faecium*	8.2	SRR32250921
EF28-PS_Lab2-raw	LAB2	*Enterococcus faecium*	9.8	SRR32250910
EF29-PS_Lab2-raw	LAB2	*Enterococcus faecium*	11	SRR32250900
EF30-PS_Lab2-raw	LAB2	*Enterococcus faecium*	5.9	SRR32250899
EF31-PS_Lab2-raw	LAB2	*Enterococcus faecium*	13	SRR32250975
EF32-PS_Lab2-raw	LAB2	*Enterococcus faecium*	6.4	SRR32250974
EF33-PS_Lab2-raw	LAB2	*Enterococcus faecium*	7.9	SRR32250973
EF34-PS_Lab2-raw	LAB2	*Enterococcus faecium*	12	SRR32250972
EF35-PS_Lab1-raw	LAB1	*Enterococcus faecium*	12	SRR31990265
EF35-PS_Lab2-raw	LAB2	*Enterococcus faecium*	13	SRR32250971
EF35-PS_Lab3-raw	LAB3	*Enterococcus faecium*	3.0	SRR31990271
EF36-PS_Lab2-raw	LAB2	*Enterococcus faecium*	7.1	SRR32250970
EF37-PS_Lab2-raw	LAB2	*Enterococcus faecium*	9.3	SRR32250969
EF38-PS_Lab2-raw	LAB2	*Enterococcus faecium*	11	SRR32250968
EF39-PS_Lab2-raw	LAB2	*Enterococcus faecium*	11	SRR32250967
EF40-PS_Lab2-raw	LAB2	*Enterococcus faecium*	11	SRR32250966
KP01-PS_Lab2-raw	LAB2	*Klebsiella pneumoniae*	14	SRR32250964
KP02-PS_Lab1-raw	LAB1	*Klebsiella pneumoniae*	24	SRR31990261
KP02-PS_Lab2-raw	LAB2	*Klebsiella pneumoniae*	15	SRR32250963
KP02-PS_Lab3-raw	LAB3	*Klebsiella pneumoniae*	14	SRR31990270
KP03-PS_Lab2-raw	LAB2	*Klebsiella pneumoniae*	16	SRR32250962
KP04-PS_Lab1-raw	LAB1	*Klebsiella pneumoniae*	52	SRR31990260
KP04-PS_Lab2-raw	LAB2	*Klebsiella pneumoniae*	19	SRR32250961
KP04-PS_Lab3-raw	LAB3	*Klebsiella pneumoniae*	13	SRR31990269
KP05-PS_Lab2-raw	LAB2	*Klebsiella pneumoniae*	18	SRR32250960
KP06-PS_Lab2-raw	LAB2	*Klebsiella pneumoniae*	27	SRR32250959
KP07-PS_Lab2-raw	LAB2	*Klebsiella pneumoniae*	19	SRR32250958
KP08-PS_Lab2-raw	LAB2	*Klebsiella pneumoniae*	24	SRR32250957
KP09-PS_Lab2-raw	LAB2	*Klebsiella pneumoniae*	23	SRR32250956
KP10-PS_Lab2-raw	LAB2	*Klebsiella pneumoniae*	27	SRR32250955
KP11-PS_Lab2-raw	LAB2	*Klebsiella pneumoniae*	24	SRR32250953
KP12-PS_Lab2-raw	LAB2	*Klebsiella pneumoniae*	17	SRR32250952
KP13-PS_Lab1-raw	LAB1	*Klebsiella pneumoniae*	39	SRR31990259
KP13-PS_Lab2-raw	LAB2	*Klebsiella pneumoniae*	14	SRR32250951
KP13-PS_Lab3-raw	LAB3	*Klebsiella pneumoniae*	38	SRR32190044
KP14-PS_Lab2-raw	LAB2	*Klebsiella pneumoniae*	19	SRR32250950
KP15-PS_Lab2-raw	LAB2	*Klebsiella pneumoniae*	17	SRR32250949
KP16-PS_Lab2-raw	LAB2	*Klebsiella pneumoniae*	15	SRR32250948
KP17-PS_Lab2-raw	LAB2	*Klebsiella pneumoniae*	16	SRR32250947
KP18-PS_Lab2-raw	LAB2	*Klebsiella pneumoniae*	17	SRR32250946
KP19-PS_Lab2-raw	LAB2	*Klebsiella pneumoniae*	15	SRR32250945
KP20-PS_Lab2-raw	LAB2	*Klebsiella pneumoniae*	15	SRR32250944
LM41-PS_Lab1-raw	LAB1	*Listeria monocytogenes*	9.5	SRR31990258
LM41-PS_Lab2-raw	LAB2	*Listeria monocytogenes*	14	SRR32250942
LM41-PS_Lab3-raw	LAB3	*Listeria monocytogenes*	34	SRR31990267
LM42-PS_Lab2-raw	LAB2	*Listeria monocytogenes*	15	SRR32250941
LM43-PS_Lab2-raw	LAB2	*Listeria monocytogenes*	11	SRR32250940
LM44-PS_Lab2-raw	LAB2	*Listeria monocytogenes*	13	SRR32250939
LM45-PS_Lab2-raw	LAB2	*Listeria monocytogenes*	11	SRR32250938
LM46-PS_Lab1-raw	LAB1	*Listeria monocytogenes*	11	SRR31990257
LM46-PS_Lab2-raw	LAB2	*Listeria monocytogenes*	12	SRR32250937
LM46-PS_Lab3-raw	LAB3	*Listeria monocytogenes*	39	SRR31990266
LM47-PS_Lab2-raw	LAB2	*Listeria monocytogenes*	9.5	SRR32250936
LM48-PS_Lab2-raw	LAB2	*Listeria monocytogenes*	16	SRR32250935
LM49-PS_Lab2-raw	LAB2	*Listeria monocytogenes*	11	SRR32250934
LM50-PS_Lab2-raw	LAB2	*Listeria monocytogenes*	11	SRR32250933
LM51-PS_Lab2-raw	LAB2	*Listeria monocytogenes*	11	SRR32250931
LM52-PS_Lab2-raw	LAB2	*Listeria monocytogenes*	7.0	SRR32250930
LM53-PS_Lab2-raw	LAB2	*Listeria monocytogenes*	13	SRR32250929
LM54-PS_Lab1-raw	LAB1	*Listeria monocytogenes*	13	SRR31990256
LM54-PS_Lab2-raw	LAB2	*Listeria monocytogenes*	9.5	SRR32250928
LM54-PS_Lab3-raw	LAB3	*Listeria monocytogenes*	43	SRR31990264
LM55-PS_Lab2-raw	LAB2	*Listeria monocytogenes*	8.3	SRR32250927
LM56-PS_Lab2-raw	LAB2	*Listeria monocytogenes*	7.8	SRR32250926
LM57-PS_Lab2-raw	LAB2	*Listeria monocytogenes*	7.3	SRR32250925
LM58-PS_Lab2-raw	LAB2	*Listeria monocytogenes*	7.8	SRR32250924
LM59-PS_Lab2-raw	LAB2	*Listeria monocytogenes*	7.5	SRR32250923
LM60-PS_Lab2-raw	LAB2	*Listeria monocytogenes*	12	SRR32250922
SA61-PS_Lab2-raw	LAB2	*Staphylococcus aureus*	11	SRR32250920
SA62-PS_Lab1-raw	LAB1	*Staphylococcus aureus*	6.7	SRR31990255
SA62-PS_Lab2-raw	LAB2	*Staphylococcus aureus*	2.7	SRR32250919
SA62-PS_Lab3-raw	LAB3	*Staphylococcus aureus*	7.0	SRR31990263
SA63-PS_Lab1-raw	LAB1	*Staphylococcus aureus*	4.5	SRR31990275
SA63-PS_Lab2-raw	LAB2	*Staphylococcus aureus*	11	SRR32250918
SA63-PS_Lab3-raw	LAB3	*Staphylococcus aureus*	19	SRR31990262
SA65-PS_Lab2-raw	LAB2	*Staphylococcus aureus*	12	SRR32250917
SA66-PS_Lab2-raw	LAB2	*Staphylococcus aureus*	9.3	SRR32250916
SA67-PS_Lab1-raw	LAB1	*Staphylococcus aureus*	4.1	SRR31990274
SA67-PS_Lab2-raw	LAB2	*Staphylococcus aureus*	12	SRR32250915
SA68-PS_Lab2-raw	LAB3	*Staphylococcus aureus*	11	SRR32250914
SA69-PS_Lab2-raw	LAB2	*Staphylococcus aureus*	14	SRR32250913
SA70-PS_Lab2-raw	LAB2	*Staphylococcus simulans*	12	SRR32250912
SA71-PS_Lab2-raw	LAB2	*Staphylococcus aureus*	13	SRR32250911
SA72-PS_Lab2-raw	LAB2	*Staphylococcus aureus*	14	SRR32250909
SA73-PS_Lab2-raw	LAB2	*Staphylococcus aureus*	9.4	SRR32250908
SA74-PS_Lab2-raw	LAB2	*Staphylococcus aureus*	9.8	SRR32250907
SA75-PS_Lab2-raw	LAB2	*Staphylococcus aureus*	11	SRR32250906
SA76-PS_Lab2-raw	LAB2	*Staphylococcus aureus*	10	SRR32250905
SA77-PS_Lab2-raw	LAB2	*Staphylococcus aureus*	7.3	SRR32250904
SA78-PS_Lab2-raw	LAB2	*Staphylococcus aureus*	8.9	SRR32250903
SA79-PS_Lab2-raw	LAB2	*Staphylococcus aureus*	7.9	SRR32250902
SA80-PS_Lab2-raw	LAB2	*Staphylococcus aureus*	6.9	SRR32250901

Each row represents a squiggle data file (uploaded as FAST5) with its corresponding library ID, identifying the file in the SRA repository. The library ID is composed of: the abbreviated species, a sequential strain numbering, ‘PS’ for performance study, the laboratory that performed the sequencing (LAB1, LAB2, LAB3), and the suffix ‘-raw’, which indicates the raw squiggle data format. For each file, we also provide the file size in gigabytes and the respective SRA accession number, which serves as a unique identifier for downloading the file, for example via the following link (https://trace.ncbi.nlm.nih.gov/Traces/?view=run_browser&display=metada).

Important note on storage format and deposition: Although the temporary FAST5 storage format has now been largely replaced by POD5, all raw signal files had to be uploaded as basecalled FAST5 files according to the NCBI SRA rules and were added to the BioProject with accession number PRJNA1091452^34^, also containing our previously published data^29^ for easy comparison. This was necessary because at the time of writing, neither SRA nor ENA accepted raw signal data in POD5 format (personal communication with the support). Our data descriptor includes scripts for the reconversion of the FAST5 files to state-of-the-art POD5 files (see below). The newly released squiggle data, provided in the temporary storage format FAST5), are labelled as “native ONT data from …” in the BioProject^34^. This distinguishes the current dataset from our previously published data in the same BioProject, where only basecalled FASTQ files were deposited, without the underlying raw signal data (squiggles) required for novel applications as presented here. For each bacterial isolate, two short-read data sets are also available from our previous study for further comparison of read accuracy^29^.

### Relation with phenotypic results for antimicrobial resistance

The Vitek test results for the phenotypic antibiotic resistance of three species — *Enterococcus faecium*, *Staphylococcus aureus*, and *Klebsiella pneumoniae —* are presented in detailed tables at Zenodo^35^. The test results include the automatic interpretation from the Vitek system and the minimum inhibitory concentration (MIC) value for 21 to 26 different antibiotics being tested, depending on the species. This additional phenotypic metadata can enhance applications of our uploaded raw signal files towards comparison of genotype versus phenotype, resistance pattern analysis, and reproducibility of nanopore long-read sequencing.

Additional material^35^ was uploaded to Zenodo to enable comprehensive sharing of unfiltered data.

## Technical Validation

We performed several validation steps to ensure the reliability and reproducibility of our raw signal data files, starting from the initial laboratory workflow related to our previous performance study, through read quality assessment, to a comprehensive validation using recognized genotyping methods in direct comparison to gold standard short-read sequencing data or between different basecalling pipelines. We further show the reproducibility of methylation detection throughout the available triplicates in our data set.

### Laboratory workflow

The validation strategy leverages the multi-laboratory technical replication framework established in the previously published performance study, ensuring robust cross-laboratory reproducibility assessment. Identical pure cultures were distributed to different laboratories in blind-coded manner for unbiased processing using the same sequencing workflow, as described in the methods. Pre-sequencing quality control involved systematic DNA concentration measurement using Qubit4 or Qubit Flex devices coupled with 1xds DNA BR or 1xds DNA HS quantification kits to ensure adequate sample concentration for downstream sequencing. After sequencing, replicates from different laboratories yielded consistent species assignments and clustered accordingly after processing through our analysis pipelines, indicating technical accuracy.

### Read statistics

The basic quality control of the basecalled reads from the released squiggle data files was carried out using NanoStat^36^ - as values reported here reflect basecalling outputs using current algorithms and may be refined through subsequent advances in basecalling software and computational models in the future. Table 4 summarizes the average values of all files per species and shows that the underlying squiggle data files are generally of good quality for nanopore reads. The quality of on average 98% of the raw reads per file is above Q10, and on average 68% of reads are above Q15, which can be significantly improved by further filtering, genome assembly, and its subsequent polishing. In addition, we achieved a high sequencing depth of 206-fold on average, as indicated by the number of bases per isolate.

**Table 4:** Read statistics of basecalled squiggle data files of all three laboratories, averaged per species.

Species	Av. N50 (bp)	Av. No. of bases per isolate	Av. Sequencing depth	Av read qual. (Q-score)
*Enterococcus faecalis*	7063	615,435,097	228	14.1
*Enterococcus faecium*	8788	501,164,127	160	14.64
*Klebsiella pneumoniae*	9751	1,192,380,320	209	15.74
*Listeria monocytogenes*	8333	799,145,316	266	15.05
*Staphylococcus aureus*	8943	518,458,520	185	15.03
*Staphylococcus simulans*	11390	686,365,684	244	14.8

The average read N50 is typically around 10 kilobases for long reads and is higher for gram-negative bacteria, such as *Klebsiella pneumoniae*, due to less disruptive DNA extraction. The number of bases indicates a high average sequencing depth, exceeding 160 times the expected genome size per species. The read quality is noted as the Q-score, which can be translated to an error rate of about 0.05% for Q15.

### Validation of long-read data in high-resolution genotyping in comparison to Illumina short-read data

**Fig. 1:**
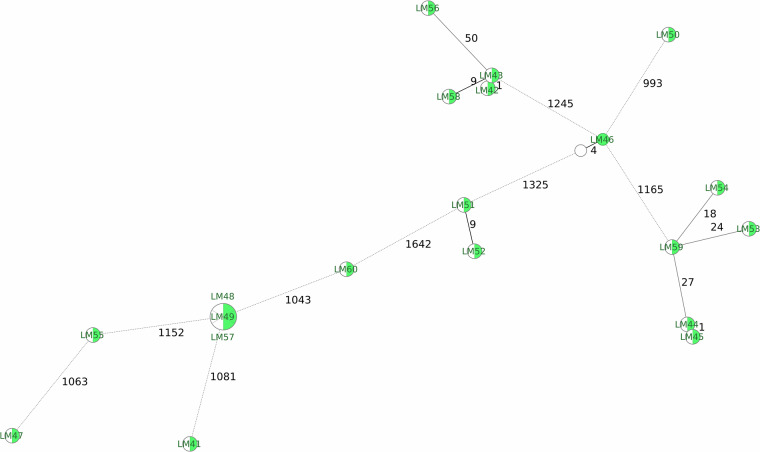
cgMLST-based minimum spanning tree, analogous to the figures in Dabernig-Heinz *et al*.^29^. The tree includes 20 different *Listeria monocytogenes* isolates, indicating the minimum distance to the nearest neighbour on the connecting lines (number of differing cgMLST targets). For each isolate, two assemblies are included, generated from either short reads (SR, in green) or long reads (LR, white) of LAB2. Except for isolate LM46 (4 mismatching targets), there are no discrepancies between LR and SR. This represents a significant improvement over our previous analysis of the same raw read files with different basecalling and pipeline versions^29^, where LM46 had 66 mismatches, and again emphasizes the importance of sharing raw signal data for re-analysis with updated software.

Next, we demonstrate the recent improvements in nanopore data compared to Illumina short-read data in a high-resolution genotyping analysis, validating the usability of our provided raw read data (Fig. 1). Such analysis, which involves re-basecalling the ONT data with the latest tool versions and models, is only possible when the raw signal data are shared. Reanalyzing the ONT data in comparison to our previous performance study indeed reveals a reduction in potentially methylation-related basecalling errors in the final assemblies, as well as a higher concordance with the short-read-based assemblies.

The pipeline consists of basecalling with SUP version 5 models (Dorado version 8.3), assembly (flye 2.9.5), and polishing (medaka 2.0.1). With this evaluation of the raw signal data shared here, we can not only demonstrate correct species assignment, but also correct sequence types and comparable assemblies in major agreement with the short-read references. The assemblies of the replicates from different laboratories demonstrate reproducibility, thereby confirming the validation of the sequencing results (Table 5). Even for previously problematic bacterial isolates that exhibited substantial typing discrepancies compared to short-read data (four strains >  = 3 mismatches, maximum of 66), newly basecalled and reanalyzed data markedly improved results (one strain >  = 3 mismatches, maximum of 4), though minor inaccuracies persisted. The later underscores the ongoing requirement for basecalling algorithm advancement, for which raw signal data (squiggles), as demonstrated and provided in this study, will be essential for evaluation and development.

**Table 5:** Genetic distance to the short-read reference of the long-read assemblies created in three different laboratories with the newest basecalling and polishing tools (dorado 0.9.0 and medaka 2.0.1).

	LAB1	LAB2	LAB3
EF22	1	0	1
EF26	1	0	0
EF35	0	0	0
KP02	0	0	0
KP04	0	0	0
KP13	0	0	0
LM41	0	0	0
LM46	2	4	0
LM54	0	0	0
SA62	0	0	0
SA63	0	0	1
SA67	0	0	0

The typing results of mostly 0 and a maximum of 4 mismatches between the complete genome assemblies and the reference represent a significant improvement compared to previously published results (maximum of 66 mismatches), which were based on the same raw squiggle data, but basecalled and assembled with older software versions (dorado 0.4.0 and medaka 1.11.3). This clearly shows the benefit of ongoing efforts to improve basecalling accuracy and sharing raw signal squiggle data.

**Fig. 2:**
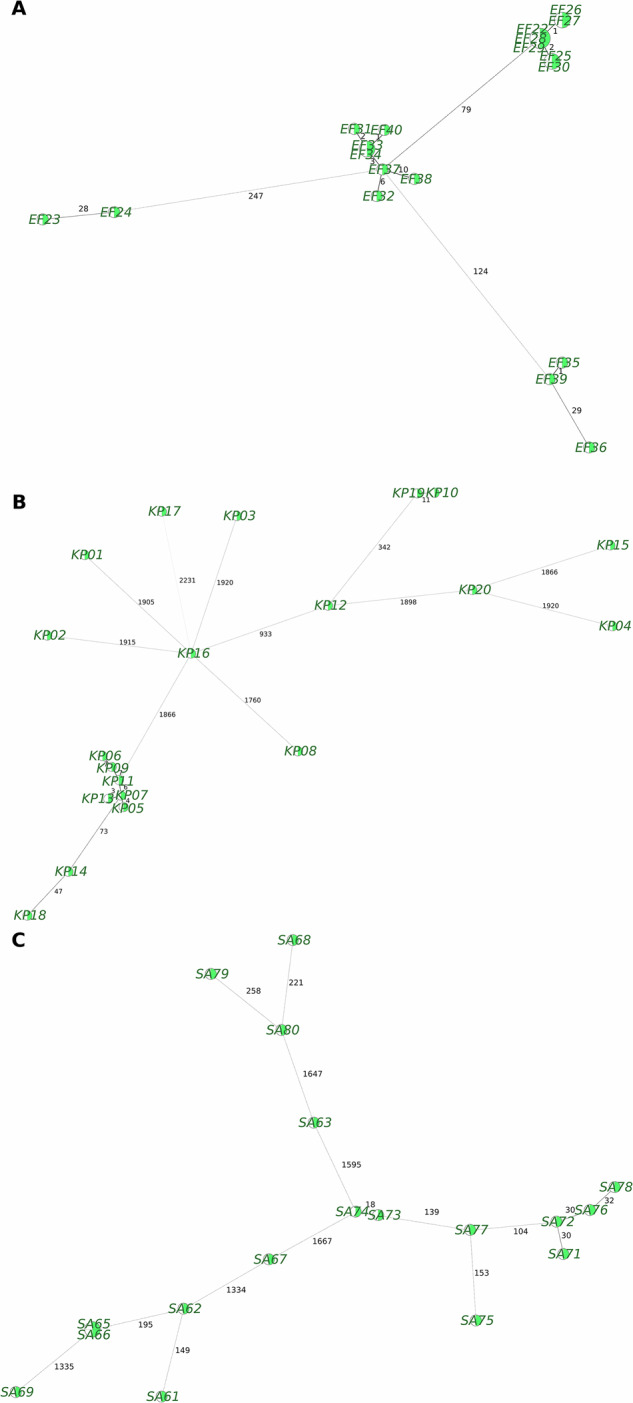
cgMLST-based minimum spanning trees, analogous to the figures in Dabernig-Heinz *et al*.^29^. These trees include both assemblies built from short reads (SR, in green) and from long reads from LAB2 (LR, white). (**A**) 19 *Enterococcus faecium*, (**B**) 20 different *Klebsiella pneumoniae* isolates and (**C**) 18 *Staphylococcus aureus*. There are no mismatches above the threshold of 3 between LR and the SR reference, except for isolate LM46 (4 mismatching targets to the reference). This is a significant improvement compared to our former analysis of the same raw read files with different basecalling versions and pipeline versions^29^.

We applied the same evaluation pipeline to all other species (Fig. 2A–C) and the replicates from the three different laboratories (Table 5). Comparing the typing results between the replicates and the short-read reference led to 0–4 mismatches in the typing results, which represents a significant improvement compared to our previously published results, which had a maximum of 66 mismatches compared to the short reads of certain isolates^29^.

### Reproducibility of methylation and motif detection

**Fig. 3:**
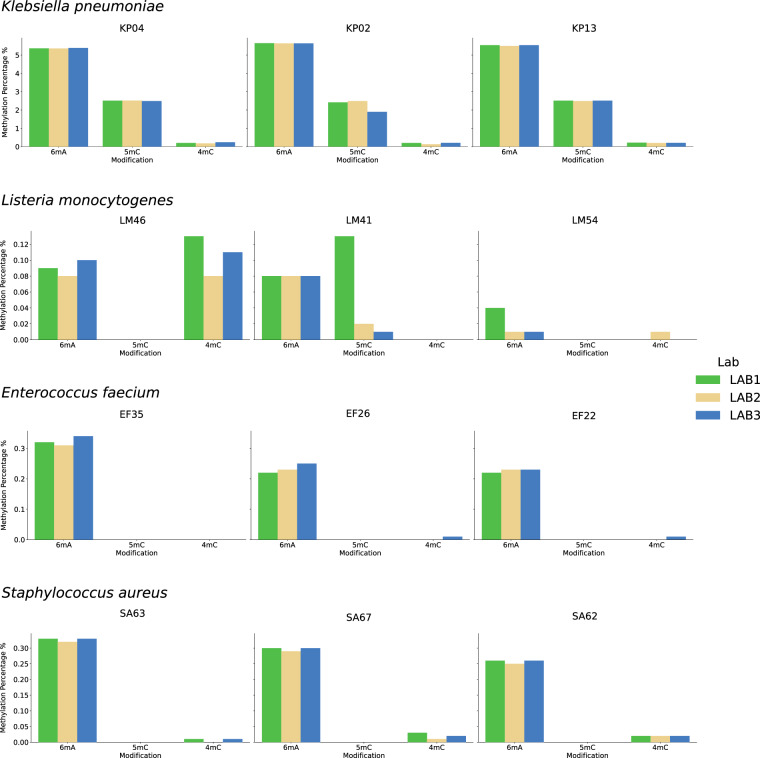
Base methylation percentages across species with multiple replicates. The percentage of methylated bases is shown for 6 mA (number of methylated adenine over total number of adenine), 5mC (methylated cytosine over total cytosine), and 4mC (methylated cytosine over total cytosine) in species with multiple replicates across all three laboratories. Replicates generally agree on methylation levels, with strong consistency for 6 mA. In contrast, 5mC and 4mC exhibit more fluctuation, particularly in *Listeria*, likely due to specific motifs that appear to be partially methylated throughout the genome.

The raw signal data from nanopore contains extensive epigenetic information, including methylation modifications such as 6 mA, 5mC, and 4mC, which are the most well-known modifications in bacterial epigenomes. To gain an overview of the methylation signals in our dataset, we ran Modkit^37^, a nanopore tool that processes raw reads containing methylation signals. After aligning the reads to a reference, the methylation level for each base (6 mA for adenine, and 5mC or 4mC for cytosine) is retrieved. We have developed and applied an in-house pipeline for preprocessing, base modification extraction, and motif detection^38^, focusing on the twelve strains with triplicate samples. Figure 3 summarizes the methylation percentages observed across replicates, showing high consistency in 6 mA detection relative to the total number of adenines. Additionally, several methylated motifs were identified with high confidence, with nearly all instances exhibiting full methylation (95–100%) in all replicates. A subset of motifs in *L. monocytogenes* was identified with only partial methylation throughout the genome (not all occurrences were methylated), which aligns with the higher fluctuations in 5mC and 4mC levels across replicates. Additionally, a specific motif in *L. monocytogenes* was found to be particularly challenging for the basecaller, as noted in both our study^32^ and another^27^, where this motif exhibited reduced accuracy in predicting methylated bases. This fluctuation suggests that potential biological or technical factors influencing methylation heterogeneity can be further investigated using our dataset. The complete list of methylated motifs and additional analyses can be found in Galeone *et al*.^32^, where we analyzed this dataset in greater detail regarding methylation.

Overall, these results highlighted the reproducibility of methylation detection analysis across the replicates. The consistent identification of methylation patterns, particularly in 6 mA, demonstrates the robustness of this approach in capturing key epigenetic modifications.

## Usage Notes

This section provides essential instructions for working with our nanopore raw signal data, including file download, conversion, basecalling, and methylation analysis.

### File Download: Exemplary from SRA

The raw signal data files provided can be downloaded using the unique accession numbers in Table 3 to search for the respective experiment in a publicly available database such as SRA. A free account registration or login might be necessary to access this database. Following this link (https://trace.ncbi.nlm.nih.gov/Traces/?view=run_browser&display=metada), the SRA numbers can be copied into the Search field. By clicking the “Search” button, the respective page of the experiment will be opened, which includes useful metadata, automated analysis pipelines, read display, and, most importantly, the data access tab. Moving to this tab, the download link to the FAST5 file is shown under “Original format”. In most browsers, this link can be used for download by clicking on it, or the URL can be used to download with “wget” using a command line interface. Due to the extensive data format, which includes preliminary basecalls as well as the raw signal data that can be further processed with various current and future basecalling models, downloading the data requires substantial storage space (~1.4 TB for all 102 files) and a suitable download speed.

### File Conversion: From FAST5 back to POD5

The raw data files were originally in POD5 format. Still, they had to be converted to the temporary storage format FAST5 for upload to the Sequence Read Archive, as SRA does not officially accept POD5 files yet (personal communication with the support). To work with the data using the latest basecalling models from ONT, the files need to be reconverted from FAST5 to POD5 after download.

To convert FAST5 files back to POD5 format, the *pod5* package^39^ provided by ONT can be used. This can be done locally via the command line:

pod5 convert from_fast5 < fast5_file > -o < output_pod5_file > , or through the web-based interface available at https://pod5.nanoporetech.com.

### Basecalling with Dorado

Once the data is converted back to POD5, we recommend using the Dorado basecaller for basecalling. A GPU is essential for optimal performance when using Dorado for basecalling, especially when using SUP models, as it significantly increases speed.

A minimal basecalling command is:


dorado basecaller sup < pod5_folder_path >  > results.bam


This command will automatically select the latest basecalling models and operate in “super accuracy” (sup) mode for the highest accuracy. For more information about Dorado basecalling and its available options, refer to the Dorado GitHub page^40^.

If simultaneous methylation analysis is desired, Dorado also supports DNA modification basecalling. The following command can be used to call methylated bases, specifically 6 mA, 5mC, and 4mC modifications:


dorado basecaller sup,6 mA,4mC_5mC < pod5_folder_path >  > results.bam


### Methylation analysis pipeline

For downstream methylation analysis, we suggest using our methylation pipeline^32^, which processes the basecalled reads generated by Dorado, including the associated methylation calls. Specifically, it extracts methylated positions along a reference genome and identifies methylated motifs using Modkit. For further details on installing and using the pipeline, please refer to the user manual on GitHub^37^.

## Supplementary information

Supplementary material

## Data Availability

All newly published files contain direct whole-genome sequencing squiggle data in pod5/fast5 format for DNA isolations from 79 bacterial strains (Bioproject Information^35^). Table 3 contains the accession numbers and file size for each squiggle data file. These files have been added to BioProject PRJNA1091452^34^, which also contains previously published data^29^. Further details on the bacterial strains in the BioProject, along with newly provided phenotypic AMR results, can be found in additional material on Zenodo^35^.

## Code availability

No custom code was used in this study.

## References

[1] Wyres, K. L., Lam, M. M. C. & Holt, K. E. Population genomics of Klebsiella pneumoniae. *Nat. Rev. Microbiol.* **18**, 344–359 (2020). 32055025 10.1038/s41579-019-0315-1

[2] Djordjevic, S. P. *et al*. Genomic surveillance for antimicrobial resistance — a One Health perspective. *Nat. Rev. Genet.* **25**, 142–157 (2024). 37749210 10.1038/s41576-023-00649-y

[3] Armstrong, G. L. *et al*. Pathogen Genomics in Public Health. *N. Engl. J. Med.* **381**, 2569–2580 (2019). 31881145 10.1056/NEJMsr1813907 PMC7008580

[4] Gardy, J. L. & Loman, N. J. Towards a genomics-informed, real-time, global pathogen surveillance system. *Nat. Rev. Genet.* **19**, 9–20 (2018). 29129921 10.1038/nrg.2017.88 PMC7097748

[5] Bogaerts, B. *et al*. Closing the gap: Oxford Nanopore Technologies R10 sequencing allows comparable results to Illumina sequencing for SNP-based outbreak investigation of bacterial pathogens. *J. Clin. Microbiol.* **62**, e01576–23 (2024). 38441926 10.1128/jcm.01576-23 PMC11077942

[6] Wick, R. R., Judd, L. M. & Holt, K. E. Assembling the perfect bacterial genome using Oxford Nanopore and Illumina sequencing. *PLOS Comput. Biol.* **19**, e1010905 (2023). 36862631 10.1371/journal.pcbi.1010905 PMC9980784

[7] Foster-Nyarko, E. *et al*. Nanopore-only assemblies for genomic surveillance of the global priority drug-resistant pathogen, Klebsiella pneumoniae. *Microb. Genomics* **9**, mgen000936 (2023). 10.1099/mgen.0.000936 PMC9997738 36752781

[8] Triebel, S. *et al*. De novo genome assembly resolving repetitive structures enables genomic analysis of 35 European Mycoplasmopsis bovis strains. *BMC Genomics* **24**, 548 (2023). 37715127 10.1186/s12864-023-09618-5 PMC10504702

[9] Zhao, W. *et al*. Oxford nanopore long-read sequencing enables the generation of complete bacterial and plasmid genomes without short-read sequencing. *Front. Microbiol*. **14** (2023). 10.3389/fmicb.2023.1179966 PMC10225699 37256057

[10] Brown, S. D., Dreolini, L., Wilson, J. F., Balasundaram, M. & Holt, R. A. Complete sequence verification of plasmid DNA using the Oxford Nanopore Technologies’ MinION device. *BMC Bioinformatics* **24**, 116 (2023). 36964503 10.1186/s12859-023-05226-y PMC10039527

[11] Sauerborn, E. *et al*. Detection of hidden antibiotic resistance through real-time genomics. *Nat. Commun.* **15**, 5494 (2024). 38944650 10.1038/s41467-024-49851-4 PMC11214615

[12] Struelens, M. J. *et al*. Real-time genomic surveillance for enhanced control of infectious diseases and antimicrobial resistance. *Front. Sci*. **2** (2024).

[13] Werner, G. *et al*. Taking hospital pathogen surveillance to the next level. *Microb. Genomics* **9**, mgen001008 (2023). 10.1099/mgen.0.001008 PMC10210943 37099616

[14] Katz, K. *et al*. The Sequence Read Archive: a decade more of explosive growth. *Nucleic Acids Res.* **50**, D387–D390 (2022). 34850094 10.1093/nar/gkab1053 PMC8728234

[15] O’Cathail, C. *et al*. The European Nucleotide Archive in 2024. *Nucleic Acids Res.* **53**, D49–D55 (2025). 39558171 10.1093/nar/gkae975 PMC11701661

[16] Wang, Y., Zhao, Y., Bollas, A., Wang, Y. & Au, K. F. Nanopore sequencing technology, bioinformatics and applications. *Nat. Biotechnol.* **39**, 1348–1365 (2021). 34750572 10.1038/s41587-021-01108-x PMC8988251

[17] Laszlo, A. H. *et al*. Decoding long nanopore sequencing reads of natural DNA. *Nat. Biotechnol.* **32**, 829–833 (2014). 24964173 10.1038/nbt.2950 PMC4126851

[18] Garalde, D. R. *et al*. Highly parallel direct RNA sequencing on an array of nanopores. *Nat. Methods* **15**, 201–206 (2018). 29334379 10.1038/nmeth.4577

[19] Martin-Baniandres, P. *et al*. Enzyme-less nanopore detection of post-translational modifications within long polypeptides. *Nat. Nanotechnol.* **18**, 1335–1340 (2023). 37500774 10.1038/s41565-023-01462-8 PMC10656283

[20] Lu, C., Bonini, A., Viel, J. H. & Maglia, G. Toward single-molecule protein sequencing using nanopores. *Nat. Biotechnol.* **43**, 312–322 (2025). 40097683 10.1038/s41587-025-02587-y PMC12006967

[21] Simpson, J. T. *et al*. Detecting DNA cytosine methylation using nanopore sequencing. *Nat. Methods* **14**, 407–410 (2017). 28218898 10.1038/nmeth.4184

[22] Stephenson, W. *et al*. Direct detection of RNA modifications and structure using single-molecule nanopore sequencing. *Cell Genomics* **2**, 100097 (2022). 35252946 10.1016/j.xgen.2022.100097 PMC8896822

[23] Bizuayehu, T. T. *et al*. Long-read single-molecule RNA structure sequencing using nanopore. *Nucleic Acids Res.* **50**, e120 (2022). 36166000 10.1093/nar/gkac775 PMC9723614

[24] Urel, H. *et al*. Nanopore- and AI-empowered metagenomic viability inference. *GigaScience*. **14**, giaf100 (2024). 10.1093/gigascience/giaf100 PMC12405693 40899150

[25] Liu-Wei, W. *et al*. Sequencing accuracy and systematic errors of nanopore direct RNA sequencing. *BMC Genomics* **25**, 528 (2024). 38807060 10.1186/s12864-024-10440-w PMC11134706

[26] Lohde, M. *et al*. Accurate bacterial outbreak tracing with Oxford Nanopore sequencing and reduction of methylation-induced errors. *Genome Res.* **34**, 2039–2047 (2024). 39443152 10.1101/gr.278848.123 PMC11610573

[27] Biggel, M., Cernela, N., Horlbog, J. A. & Stephan, R. Oxford Nanopore's 2024 sequencing technology for Listeria monocytogenes outbreak detection and source attribution: progress and clone-specific challenges. *J. Clin. Microbiol.* **62**, e01083–24 (2024). 10.1128/jcm.01083-24 PMC11559035 39365069

[28] Linde, J. *et al*. Comparison of Illumina and Oxford Nanopore Technology for genome analysis of Francisella tularensis, Bacillus anthracis, and Brucella suis. *BMC Genomics* **24**, 258 (2023). 37173617 10.1186/s12864-023-09343-z PMC10182678

[29] Dabernig-Heinz, J. *et al*. A multicenter study on accuracy and reproducibility of nanopore sequencing-based genotyping of bacterial pathogens. *J. Clin. Microbiol.* **62**, e00628–24 (2024). 39158309 10.1128/jcm.00628-24 PMC11389150

[30] nanoporetech/medaka: Sequence correction provided by ONT Research. https://github.com/nanoporetech/medaka.

[31] Wick, R. Medaka v2: progress and potential pitfalls. *Ryan Wick's bioinformatics blog* https://rrwick.github.io/2024/10/17/medaka-v2.html (2024).

[32] Galeone, V. *et al*. Decoding bacterial methylomes in four public health-relevant microbial species: nanopore sequencing enables reproducible analysis of DNA modifications. *BMC Genomics* **26**, 394 (2025). 40269718 10.1186/s12864-025-11592-z PMC12016153

[33] Samarakoon, H. *et al*. Flexible and efficient handling of nanopore sequencing signal data with slow5tools. *Genome Biol.* **24**, 69 (2023). 37024927 10.1186/s13059-023-02910-3 PMC10080837

[34] *NCBI Sequence Read Archive* https://identifiers.org/ncbi/insdc.sra:SRP497546 (2024)

[35] Dabernig-Heinz, J. A whole-genome sequencing dataset of nanopore raw signals for bacterial genotyping and methylation analysis - further information on AMR and BioProject. *Zenodo*10.5281/zenodo.17542640 10.1038/s41597-025-06319-4 PMC12675498 41331261

[36] Lee, S. C.-H. & Burke, P. J. NanoStat: An open source, fully wireless potentiostat. *Electrochimica Acta* **422**, 140481 (2022).

[37] nanoporetech/modkit. Oxford Nanopore Technologies (2025).

[38] rki-mf1/ont-methylation. RKI MF1 Bioinformatics (2025).

[39] nanoporetech/pod5-file-format. Oxford Nanopore Technologies (2025).

[40] nanoporetech/dorado. Oxford Nanopore Technologies (2025).

