# Supplementary material for: A whole-genome sequencing dataset of nanopore raw signals for bacterial genotyping and methylation analysis
Source: Sci Data. 2025 Dec 2;12:1905. doi: 10.1038/s41597-025-06319-4 (PMC12675498; doi:10.1038/s41597-025-06319-4)
Supplement: Supplementary file 1 — Supplementary material [file 41597_2025_6319_MOESM1_ESM.docx]

### Supplementary Material

**Supplementary Table 1**: Newly published, detailed characteristics of the nanopore sequencing performed in our previous multi-centre performance study, analogous to Table 2 in the main text. All characteristics of the sequencing runs are averages or sums of all 4 to 6 runs (number of flow cells used). The DNA amount loaded onto the flow cell after library preparation is specified either in femtomole or in absolute nanograms of input DNA. Typically, 10 to 20 strains of the same or similar species were pooled via barcoding in a shared library. The produced data comprised FAST5 files for the discontinued 260 bp/s sequencing mode.

|  | **Nanopore Sequencing (SQK-NBD114.24, R10.4.1, 260 bp/s, 4 kHz)** | | | | | | | | |
| --- | --- | --- | --- | --- | --- | --- | --- | --- | --- |
|  | **DNA amount loaded (ng)** | **Samples per run** | **MinKNOW version** | **Average pores before sequencing** | **Number of used flowcells** | **Data produced (GB)** | **Bases sequenced (Gb)** | **N50 (kb)** | **Fail rate (%)** |
| LAB1 | 150 | 20 | 22.12.7 | 1425 | 5 | 876 | 52.94 | 9.7 | 15.6 |
| LAB2 | 90 | 20 | 22.12.7 | 1500 | 4 | 940 | 57.31 | 8.3 | 17.5 |
| LAB3 | 114 - 1130.4 | 20 | 22.10.5 - 22.12.5 | 1489 | 6 | 1494 | 79.27 | 7.5 | 15.6 |
| LAB4 | 50-55 | 10 - 20 | 22.12.5 | 1438 | 6 | 730 | - | - | - |
| LAB5 | 20 fmol | 10 - 20 | 22.10.5 | 1349 | 4 | 1129 | - | 14.8 | - |

### 
